# Host Genetic Effects and Phenotypic Landscapes of Rumen Bacterial Enterotypes in a Large Sheep Population

**DOI:** 10.3390/ani15182724

**Published:** 2025-09-17

**Authors:** Yukun Zhang, Fadi Li, Xiaoxue Zhang, Deyin Zhang, Weimin Wang

**Affiliations:** 1 State Key Laboratory of Herbage Improvement and Grassland Agro-Ecosystems, Key Laboratory of Grassland Livestock Industry Innovation, Ministry of Agriculture and Rural Afairs, Engineering Research Center of Grassland Industry, Ministry of Education, College of Pastoral Agriculture Science and Technology, Lanzhou University, Lanzhou 730020, China; zhangyukun.lzu@foxmail.com (Y.Z.); lifd@lzu.edu.cn (F.L.); zdy1213@163.com (D.Z.); 2 College of Animal Science and Technology, Gansu Agricultural University, Lanzhou 730046, China; zhangxx@gsau.edu.cn

**Keywords:** sheep, rumen microbiome, enterotype, phenotypic landscapes, host genetic effects

## Abstract

Population stratification based on gut microbiota composition has revealed several enterotypes in humans and animals, providing valuable tools for studying the gut microbiota landscape, which is crucial for animal health and production. However, knowledge about rumen enterotype identification in sheep, its influencing factors, and its association with growth performance and host genetics remains limited. Here, we investigated host genetic effects and phenotypic landscapes of rumen bacterial enterotypes in a large sheep population. Ruminal contents from 1150 healthy sheep were analyzed using 16S rRNA gene sequencing and genus-level clustering, complemented by extensive phenotypic data covering 47 traits spanning growth, feed efficiency, meat yield, and ruminal fermentation, along with whole-genome resequencing data. We identified two distinct enterotypes: Enterotype 1 (E1), a mixture of multiple genera, and Enterotype 2 (E2), dominated by *Prevotella*. E2 sheep exhibit superior growth and meat production performance, but lower feed efficiency and increased fat deposition. Two-part beta-regression models and co-occurrence network analyses revealed the extensive impact of enterotypes on microbial community structure, with E1 displaying a higher frequency of unique bacterial interactions. The estimated heritability of the enterotype was 0.47, and a GWAS identified five key genetic markers associated with rumen enterotype, localized to two candidate genes: *CHODL* and *ENPP6*. These markers significantly influence 58 ruminal bacterial genera, including key taxa and driving genus. Overall, our data provide new insights into sheep rumen-enterotype characteristics, contributing to a better understanding of microbial interactions that are crucial for improving ruminant growth performance.

## Simple Summary

The complex interactions between host animals and their gut microbiota offer new pathways for improving energy utilization and production efficiency in livestock farming. Over a decade after its introduction, the concept of enterotype continues to be studied and expanded into areas such as human microbiota types (e.g., vaginal type, oral type) and environmental types (e.g., soil type, marine type). In this study, we applied this concept to the rumen microbiota of sheep. By analyzing the rumen microbiota of 1150 sheep, we identified two distinct rumen microbiota types, E1 and E2, and examined their effects on various traits. Sheep with the E2 demonstrated better growth and meat quality, but lower feed efficiency and higher fat deposition. The rumen enterotype is influenced by the sheep’s genome, and we identified five genomic markers associated with enterotype that affect the composition of specific rumen microbiota.

## 1. Introduction

Sheep (*Ovis aries*), among the first domesticated ruminants, have been selectively bred for wool, meat, dairy, and hides [1,2]. Rumen, home to a diverse microbial community, is essential for the sheep’s metabolic functions, including immunity, development, and nutrition [3,4]. Rumen microbiota ferment plant material into volatile fatty acids (VFA), which provide 70–80% of the sheep’s energy, directly influencing its productivity [5,6,7,8]. Recent advances in high-throughput sequencing have highlighted the dynamic nature of the rumen microbiome and its crucial role in sheep growth and health [9]. Understanding this microbiome is key to developing strategies that enhance sheep productivity and overall well-being.

With the paradigm shift in microbiome research, Arumugam et al. [10] defined the concept of enterotype within the human gut microbiome, referring to ecological clusters formed by dense groupings of samples in a multidimensional space based on the gut microbial community composition. Analysis of 33 human fecal samples from different populations revealed that the human gut microbiome can be classified into three predominant enterotypes, primarily dominated by *Bacteroides*, *Prevotella*, and *Ruminococcus* [10]. Due to its ability to simplify the complexity of microbiome data [11], the enterotype concept has been widely applied in human disease diagnostics and animal productivity research. Enterotyping of human fecal samples showed that bacteria like *Prevotella* and *Bifidobacterium* were mainly found in healthy individuals, but not consistently in those with diabetes [12]. In pigs, fecal microbiome enterotypes are linked to factors such as age, gender, diet, breed, and health [13,14,15,16]. Studies in dairy cattle have demonstrated a clear correlation between fecal microbiome enterotypes and the animal’s health, immunity, and productivity. Three enterotypes have been identified, controlled by *Bifidobacteria*, unclassified *Clostridia*, and unclassified *Spirillaceae*. Animals in the *Bifidobacteria* enterotype exhibited superior milk quality, lower body weight, fewer health issues, and a reduced risk of ketosis [17]. A study integrating rumen and fecal microbiomes of 308 dairy cows revealed distinct microbial communities in each compartment, further divided into two enterotypes [18]. Notably, cows with a *Prevotella*-dominated rumen microbiome displayed significantly better milk production performance, whereas no similar production efficiency differences were observed between the two fecal microbiome-based enterotypes [18]. This finding emphasizes the critical role of rumen enterotypes in optimizing ruminant nutritional management. However, research on rumen enterotypes in ruminants remains in its infancy, predominantly focused on dairy cattle [17,18,19,20], with limited understanding of the rumen microbiome enterotypes in sheep and their potential impact on individual sheep productivity. Although we previously characterized the enterotypes of the whole gut microbiomes of 36 sheep, the sample size was small and lacked sufficient population diversity, limiting the ability to accurately depict the enterotype spectrum [21]. Studies based on large-scale animal populations will be instrumental in deepening our understanding of the systemic role of rumen enterotypes in sheep productivity [22].

The host genetic influence on gut microbiota remains a topic of ongoing debate. Recently, INRAE researchers bred two pig lines, each selected for one of two distinct gut microbiota enterotypes: the PM enterotype driven by *Prevotella* and *Mitsuokella*, and the RT enterotype driven by *Ruminococcus* and *Treponema*. Tracking the gut microbiota of piglets across three generations revealed a significant increase in the frequency of the target enterotype in both lines, from 53% to 87% in the PM line and from 47% to 70% in the RT line [23]. Our previous research identified the α and β diversity of bacterial communities in the sheep rumen microbiota, as well as 52 bacterial genera exhibiting significant heritable traits [22]. These findings underscore the pivotal role of host genetics in shaping the composition of gut microbiota and suggest that such genetic factors may influence the formation and stability of enterotypes. However, our current understanding of the heritability and the key genes involved in regulating the rumen microbiome enterotypes in sheep is lacking. In this study, our objective is to utilize a large-scale sheep population as a ruminant model to investigate the differences in rumen microbial communities and their association with host growth traits, while also exploring the influence of host genetics on the assembly of rumen microbial enterotypes. This will elucidate the systematic role of rumen enterotypes in sheep production systems and provide guidelines for the development of breeding strategies aimed at modulating rumen microbiome composition.

## 2. Materials and Methods

### 2.1. Animals and Sample Collection

**Figure 1:**
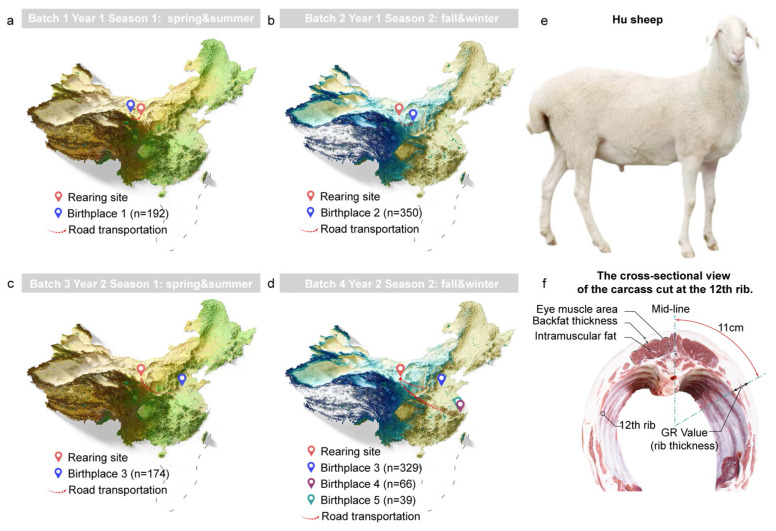
Photos of the animals and their carcass cuts. (**a**–**d**) The batches, rearing years, rearing seasons and birthplace of large-scale sheep populations. (**e**) A photo of a male Hu sheep. (**f**) The transverse cross-section of the 12th rib of a sheep’s carcass.

A total of 1150 healthy male Hu lambs were randomly chosen and reared under identical feeding conditions in the study. These sheep were reared in four distinct batches over a two-year period, with one batch in the spring/summer and another in the fall/winter each year (Figure 1a–e). The specific details are as follows: Batch 1 (spring and summer of Year 1): 192 animals sourced from Jinchang Zhongtian Sheep Industry Co., Ltd. (Birthplace 1 (Jinchang, China)). Batch 2 (fall and winter of Year 1): 350 male lambs from Huanxian Zhongsheng Sheep Industry Development Co., Ltd. (Birthplace 2 (Qingyang, China)). Batch 3 (spring and summer of Year 2): 174 male lambs from Linqing Runlin Animal Husbandry Co., Ltd. (Birthplace 3 (Liaocheng, China)). Batch 4 (fall and winter of Year 2): 434 male lambs, including 329 from Linqing Runlin Animal Husbandry Co., Ltd., 66 from Hangzhou Pangda Agricultural Development Co., Ltd. (Birthplace 4 (Hangzhou, China)), and 39 from Changxing Yongsheng Animal Husbandry Co., Ltd. (Birthplace 5 (Hangzhou, China)). Lambs were transferred with their dams from the lambing pen to a suckling pen at 3–4 days of age and remained there until weaning at 56 days of age. During this period, the lambs were entirely dependent on maternal milk, without any feed intake, and had free access to water. The indoor temperature was maintained at approximately 25 °C.

At 56 days of age, the lambs were weaned artificially and transported to the Minqin Experimental Farm of Lanzhou University for individual pen feeding, where they received the same diet (Table S1) until slaughter at 180 days of age. To be more precise, the lambs underwent a 14-day acclimatization period during which the proportion of pelleted feed in the diet was incrementally increased by 7.1% daily, with a corresponding decrease in the proportion of hay, until the lambs consumed only pelleted feed. This was followed by a 10-day preliminary trial period and a 100-day formal trial period, throughout which all the sheep were provided with identical pellet feed and had ad libitum access to feed and water. The rearing conditions and environment were standardized throughout the entire experiment. After a 12-h fast and at the conclusion of the experiment (at 180 days of age), blood samples (5 mL; one sample per animal) were collected from the jugular vein in the morning under the supervision of a qualified veterinarian for subsequent DNA extraction. All sheep were humanely slaughtered in a licensed slaughterhouse by a veterinarian, following ethical guidelines to ensure humane treatment. After confirming death, the veterinarian opened the abdominal cavity and removed the entire rumen. Using sterile scissors (Gansu Tecovi Bio-technology Co., Lanzhou, China), small incisions were then made in specific regions of the rumen: the dorsal sac, ventral sac, dorsal blind sac, and ventral blind sac. sterile scissors. The rumen contents were collected, thoroughly mixed, and then filtered through four layers of cheesecloth (Gansu Tecovi Bio-technology Co., Lanzhou, China) to separate the liquid fraction. The liquid was subsequently transferred and equally divided into two sterile centrifuge tubes (Thermo Fisher Scientific Inc., Waltham, MA, USA). One portion was preserved at a low temperature of −80 °C for 16S rRNA sequencing; the other portion of the rumen fluid was mixed with 25% phosphoric acid (Sinopharm Chemical Reagent Co., Ltd., Shanghai, China) in a ratio of 9:1 (*v*/*v*), placed into a centrifuge tube, and stored at −20 °C for the determination of VFAs. In addition, a 1 cm^2^ sample of the rumen ventral sac tissue was collected and preserved in 4% formaldehyde (Sinopharm Chemical Reagent Co., Ltd., Shanghai, China) solution for the subsequent preparation of histological sections.

### 2.2. Animal Performance, Ruminal Fermentation, and Rumen Development Parameters

All the lambs were fed individually, with each lamb confined to a separate pen. At 8:00 a.m. on day 0 (80 days of age) and day 100 (180 days of age) of the experimental recording period, the lambs, which had been fasted for 12 hours, were weighed using a veterinary electronic scale (Gansu Tecovi Bio-technology Co., Lanzhou, China). The body length and chest circumference of all the sheep were measured with a soft tape measure (Gansu Tecovi Bio-technology Co., Lanzhou, China), and the body mass index (BMI = body weight (kg)/[body length (m)]^2^) were calculated [24]. In addition, the remaining feed quantity in the bunk was weighed before each feeding to calculate the total feed intake (FI), average daily feed intake (ADFI; ADFI = FI/N, where N represents the number of days), feed conversion ratio (FCR = ADFI/ADG, where ADG is the average daily gain), and residual feed intake (RFI). The RFI was calculated using the following formula [25]: Y_j_ = β_0_ + β_1_MBW_j_ + β_2_ADG_j_ + e_j_, where MBW is mid-test metabolic weight (MBW = [0.5 * (BW80 + BW180)]^0.75^), ADG = (180-day weight − 80-day weight)/N. **Y_j_** represents the ADFI of the jth individual; β_0_ is the regression intercept; β_1_ is the regression coefficient of MBW; **β_2_** is the regression coefficient of ADG; **e_j_** is the non-controllable error of the **jth** individual; and N is the experimental period (100 days).

Immediately after slaughter, the skin and wool, head, forelimbs below the carpal joint, hind limbs below the tarsal joint, and internal organs (with the kidneys and renal fat retained) were promptly excised and removed. The carcass was then weighed, and the dressing percentage (carcass weight/pre-slaughter live weight) was calculated. Subsequently, the tail fat, perirenal fat, and omental fat were immediately separated and individually weighed using an electronic scale to obtain their respective weights. The relative weight of tail fat (based on carcass weight) was calculated by dividing the absolute weight of tail fat by the carcass weight. The relative weight of tail fat (based on pre-slaughter live weight) was equal to the absolute weight of tail fat divided by the live weight. Similarly, we calculated the relative weights of perirenal and mesenteric fat with respect to live weight. The relative weight of total fat (based on pre-slaughter live weight) was equal to the sum of the weights of tail fat, perirenal fat, and omental fat divided by the pre-slaughter live weight. After the carcass was cooled in a refrigerated room (at 4 °C) for 12 h to allow for acid draining, it was horizontally cut at the posterior edge of the 12th rib (Figure 1f). Then, a sheet of sulfite paper was placed on the cross-section of the eye muscle, and the outline of the eye muscle cross-section was traced with a soft pencil. The sulfite paper was scanned using an EPSON color high-speed scanner (Perfection V850 Pro; Seiko Epson Corporation, Suwa, Japan), and the area of the eye muscle (EMA) was calculated using ImageJ software (ij154-win-java8). Here, we also measured the backfat thickness and Greville (GR value, also called rib thickness) tissue depth [26]. The method for measuring backfat thickness involved horizontally cutting the carcass at the posterior end of the 12th rib and using a vernier caliper (Gansu Tecovi Bio-technology Co., Lanzhou, China) to measure the thickness of the fat layer directly above the midpoint of the eye muscle between the 12th and 13th ribs. The GR value was assessed by measuring the tissue thickness at a point 11 cm away from the midline at the intersection of the 12th and 13th ribs using a vernier caliper. All procedures for separating fat tissues were carried out by a certified animal-handling veterinarian. In addition, we used a near-infrared transmission spectrometry meat composition analyzer (FOSS FoodScan™-1, Hillerød, Denmark) to determine the meat quality of the Longissimus dorsi muscle. Specifically, we collected 100 g of the to-be-tested meat from the same location of the Longissimus dorsi muscle, removing the surface fascia and fat. The meat to be tested was minced using a meat grinder, and three biological replicate samples were taken for each sample, with two additional technical replicate samples collected for each biological replicate. Subsequently, they were placed in Petri dishes, spread out, and pressed onto the surface. Then, a FoodScan Meat Analyzer (FOSS FoodScan™-1, Hillerød, Denmark) was used to determine the contents of water, protein, intramuscular fat, collagen and salt.

The rumen fluid preserved with phosphoric acid was thawed and subsequently centrifuged at 4 °C for 10 min at 12,000× *g*. In accordance with our previously established method [6], gas chromatographic analysis was conducted on a Thermo Fisher Trace 1300 gas chromatography system (Thermo Scientific, TRACE 1300, Milan, Italy) equipped with a DB-FFAP capillary column (15 m × 0.32 mm × 0.25 µm) to quantify the concentration of VFAs in the rumen fluid. We utilized 1-centimeter-square rumen tissue preserved in 4% formaldehyde solution and followed the paraffin section-making procedure of Xue et al. to prepare rumen tissue sections [27]. After paraffin embedding, sectioning, and hematoxylin-eosin staining, 3 to 5 sections were selected, and 5 typical fields (with intact tissue) were chosen from each section. The Image-Pro Express 6.0 image analysis system software was employed to measure the length and width of the rumen papillae in the rumen ventral sac as well as the thickness of the rumen muscle layer.

### 2.3. 16S rRNA Gene Sequencing and Analysis

Each rumen content sample was individually thawed on ice and then homogenized. Subsequently, total microbial DNA was extracted from approximately 200 mg of each sample using the EasyPure Stool Genomic DNA Kit (TransGen Biotech, EE301-01, Beijing, China) in accordance with the manufacturer’s instructions. The V3-V4 region of the bacterial 16S rRNA gene was amplified using barcoded primers (341F: CCTAYGGGRBGCASCAG and 806R: GGACTACNNGGGTATCTAAT). Amplicon sequencing was performed on the NovaSeq PE250 platform at Illumina (Novogene Biotech Co., Ltd., Beijing, China). Raw sequences were assigned to samples based on their unique barcodes and then trimmed to remove the barcode and primer sequences. Paired-end reads of each sample were assembled using FLASH (Version 1.2.11) software [28]. Clean sequences were subjected to quality control analysis using the FastQC (Version 0.11.9) [29] software, and chimeric sequences were removed using UCHIME (Version 4.2.40) [30]. The filtered data were further processed in QIIME2 (Version 2021.11) [31] using the DADA2 method [32] to generate an amplicon sequence variant (ASV) table. A rarefied ASV count table was generated using the QIIME2 *feature-table rarefy* command and the minimum library size method, incorporating data from 11,976 ASVs and yielding 813 genera. Taxonomic assignment was performed using the QIIME2 *classify-sklearn* algorithm with a pre-trained Naive Bayes classifier on the 16S Silva database (Version 138) [6]. The alpha diversity metrics, including Fisher’s α, Chao1, InvSimpson (Inverse Simpson Index), Observed, Pielou’s Evenness, Abundance-based Coverage Estimator (ACE), Shannon–Wiener Index, and Simpson’s Index, were calculated using the *Vegan* package (Version 2.6-2) [33] in R. Distance calculations for beta diversity, including principal coordinate analysis (PCoA) and non-metric multidimensional scaling (NMDS), were performed in the *Microeco* (Version 1.14.0) R package [34] based on the Bray–Curtis method.

The enterotypes were determined according to the standard method described by Arumugam et al. [10]. The driver bacterial genera for each enterotype were identified using a random forest model based on the *randomForest* (Version 4.6-14) [35] R package with 10-fold cross-validation repeated 999 times.

### 2.4. Analysis of Enterotype-Covariate Links and Covariate Collinearity

We initially conducted Permutational Multivariate Analysis of Variance (PERMANOVA) using the *adonis2* function from the *vegan* (Version 2.6-2) [33] package in R to assess the impact of four covariates (Batch, Birthplace, Rear year, and Season) on rumen microbial genera. We constructed the model based on the Bray–Curtis distance matrix and performed 999 permutations to determine the significance of the covariates’ effects on microbial community structure. Then, we performed chi-square tests using the *chisq.test* function from the *stats* R package (V 4.4.1) [36] to assess the overall impact of four covariates on enterotype distribution. Subsequently, pairwise chi-square tests were conducted using the *pairwise.prop.test* function to identify which specific groups exhibited significant differences. Finally, logistic regression models were fitted using the *glm* function, and the effects of the covariates, while controlling for other variables, were evaluated using the summary function. The specific models included: the effect of Batch on enterotype distribution (Model 1), the effect of Birthplace on enterotype distribution (Model 2), the effect of Rear year on enterotype distribution (Model 3), and the effect of Season on enterotype distribution (Model 4). We also calculated the condition index (CI) using the *kappa* function to assess multicollinearity among multiple covariates. Additionally, we used the *ppcor* package (V 1.1) [37] to compute Spearman’s rank correlations to further analyze the nonlinear relationships among covariates. The condition index, a statistic based on the eigenvalues of the design matrix, quantifies the degree of linear dependence among covariates.

### 2.5. Linear Regression Model for Comparing Animal Phenotypes Across Enterotypes

To investigate the association between enterotype and animal phenotypes, we employed a linear regression model. The model was designed to assess the impact of enterotype on phenotypic traits while controlling for potential confounding variables. The linear regression model was specified as follows:

(1) $$y_{i}={\beta_{0}+\beta }_{1}{Enterotype}_{i}+\beta_{2}{Birthplace}_{i}+\epsilon_{i}$$

where $y_{i}$ represents the phenotypic trait for the i-th animal; $\beta_{0}$ is the intercept term; $\beta_{1}$ is the regression coefficient for enterotype, indicating the effect of enterotype on the phenotype; $\beta_{2}$ is the regression coefficient for birthplace, included as a fixed effect to control for potential confounding due to geographical origin. ${Enterotype}_{i}$ is a categorical variable representing the enterotype of the i-th animal. ${Birthplace}_{i}$ is a categorical variable representing the birthplace of the i-th animal. $\epsilon_{i}$ is the error term, assumed to be normally distributed with mean 0 and constant variance $\sigma^{2}$. The model was fitted using ordinary least squares regression. The significance of the regression coefficients was assessed using *t*-tests. Specifically, we focused on the *p*-values associated with the enterotype coefficients ($\beta_{1}$) to evaluate the significance of the enterotype effect on the phenotypic traits. To control the false discovery rate (FDR), *p*-values were adjusted using the Benjamini–Hochberg (BH) method.

### 2.6. Microbiota Differences Across Distinct Enterotypes

The relationship between enterotypes and the rumen microbiome was investigated by first analyzing the correlation between the alpha diversity index and enterotype using the same linear regression model described previously (Formula (1)). Next, we used Linear Discriminant Analysis Effect Size (LEfSe) to investigate the differential microbiota at the full taxonomic level. Furthermore, genus-level analyses were conducted using a regression model, employing a two-part model to account for the zero-inflated nature of microbial data. Data can be divided into two parts based on the prevalence of each genus in the population (the proportion of non-zero values). For the first part, targeting bacteria with a prevalence greater than 60% [22], we employed Beta regression. Details of the two-part model are given below:

(2) $$logit\left(\mu_{i}\right)={\beta_{0}+\beta }_{1}{Enterotype}_{i}+\beta_{2}{Birthplace}_{i}$$

In the model, $\mu_{i}$ is the mean of the i-th observation, and the logit link function is defined as $logit\left(\mu_{i}\right)=log(\frac{\mu_{i}}{1-\mu_{i}})$. The regression coefficients are $\beta_{0}$, $\beta_{1}$, and $\beta_{2}$, where $\beta_{0}$ is the intercept, $\beta_{1}$ is the coefficient for Enterotype, and $\beta_{2}$ is the coefficient for Birthplace. ${Enterotype}_{i}$ and ${Birthplace}_{i}$ represent the enterotype and birthplace of the i-th observation, respectively. Assuming $y_{i}$ follows a Beta distribution, its probability density function is given by $f\left(y_{i};\alpha ,\beta \right)=\frac{y_{i}^{\alpha -1}{\left(1-y_{i}\right)}^{\beta -1}}{B(\alpha ,\beta )}$, where α and β are the shape parameters of the Beta distribution, and B(α,β) is the Beta function.

For the second part, targeting bacteria with a prevalence less than 60%, we utilized Zero-Inflated Beta Regression (ZIBR). The model consists of two parts: the zero-probability part and the mean part.

1. Zero-Inflation Component:

(3) $$\mathrm{logit}\left(v_{i}\right)=\gamma_{0}+\gamma_{1}{Enterotype}_{i}+\gamma_{2}{Birthplace}_{i}$$

2. Mean Component:

(4) $$logit\left(\mu_{i}\right)={\beta_{0}+\beta }_{1}{Enterotype}_{i}+\beta_{2}{Birthplace}_{i}$$

where $v_{i}$ is the probability of the i-th observation being zero. $\mu_{i}$ is the mean of the i-th observation. $logit\left(v_{i}\right)=log(\frac{v_{i}}{1-v_{i}})$ and $logit\left(\mu_{i}\right)=\log\left(\frac{\mu_{i}}{1-\mu_{i}}\right)$ are the logit link functions. $\gamma_{0}$, $\gamma_{1}$, and $\gamma_{2}$ are the regression coefficients for the zero-inflation component. $\beta_{0}$, $\beta_{1}$, and $\beta_{2}$ are the regression coefficients for the mean component. ${Enterotype}_{i}$ and ${Birthplace}_{i}$ are the enterotype and birthplace of the i-th observation, respectively. It is assumed that $y_{i}$ follows a Zero-Inflated Beta distribution with the probability density function:

(5) $$f\left(y_{i};\mu_{i},v_{i},\phi \right)=\left\{\begin{array}{c} v_{i},\;\mathrm{if}\;y_{i}\;=\;0\\ 1-v_{i}\cdot \frac{y_{i}^{\mu_{i}\phi -1}{\left(1-y_{i}\right)}^{\left(1-\mu_{i}\right)\phi -1}}{B\left(\mu_{i}\phi ,\left(1-\mu_{i}\right)\phi \right)},\;\mathrm{if}\;y_{i}>0 \end{array}\right.$$

where ϕ is the precision parameter that controls the shape of the distribution, and $B\left(\mu_{i}\phi ,\left(1-\mu_{i}\right)\phi \right)$ is the Beta function. These models were fitted using the *gamlss* function from the *gamlss* package (V 5.4-22) [38]. The significance of the regression coefficients was assessed using *t*-tests. Specifically, we focused on the *p*-values associated with the enterotype coefficients ($\beta_{1}$) to evaluate the significance of the enterotype effect on microbiota abundance. To control the FDR, *p*-values were adjusted using the BH method. An FDR threshold of ≤0.05 was employed to identify bacterial genera that significantly influence enterotype.

### 2.7. Microbial Co-Occurrence Network Analysis

The microbial co-occurrence network was constructed using Spearman partial correlation in different enterotypes, using the R package *ppcor* (Version 1.1) [37]. To reduce noise and the false positive rate, only genera that were present in at least 50% of the samples were included in the network, with the additional criterion that each genus had an average relative abundance greater than 0.001. In each microbial network, correlations with adjusted *p*-values greater than 0.05 were filtered out using FDR correction based on the BH method. The *vegan* [33] and *igraph* (Version 2.1.4) [39] packages were used to evaluate various network topological parameters, including the number of vertices, number of edges, clustering coefficient, average distance, and average separation [40]. We defined edges that only appeared in one enterotype network as specialist edges, and edges that appeared in all two enterotype networks as generalist edges. For the pairs of relationships that are present in both enterotypes, i.e., generalist edges of co-occurrence network, we further assessed whether their correlations changed or remained stable across the two enterotypes. Based on a comparative analysis of the correlation coefficients, we categorized the generalist relationship pairs into three types: those with directional changes, those with changes in intensity, and those that are stable. If a pair exhibits a positive correlation in E1 (Correlation coefficient greater than 0) and a negative correlation in E2 (Correlation coefficient is less than 0), or vice versa, it is considered a change in the direction of correlation. Even when the direction remains the same, if the absolute value of the correlation coefficient significantly differs (with a difference greater than 0.2), the strength of the correlation is deemed to have changed. If the correlation coefficients for a pair of bacteria exhibit consistent direction and strength in both E1 and E2, it is classified as a stable correlation. Key taxa are defined as those microbial taxa with high connectivity within the microbial community, and they serve as drivers of microbial community structure and function [40,41]. In addition, we also calculated the number of positive and negative correlation types in the two enterotype networks to assess the complexity of the microbial network.

### 2.8. Genotyping and Quality Control

Host genomic DNA was extracted from blood samples using the EasyPure Blood Genomic DNA Kit (Tiangen Biotech Co., Ltd., Beijing, China). The quality of the DNA was assessed by 1% agarose gel electrophoresis (Sigma-Aldrich, Shanghai, China). A total of 1150 qualified host DNA samples were subjected to whole-genome re-sequencing on the Illumina Hiseq Xten platform (PE150) with an average depth of approximately 7×. Variant calling for all samples was performed following a standardized bioinformatics pipeline [42]. Specifically, each DNA sample was randomly fragmented into 350 bp fragments using a Covaris sonicator, followed by library preparation and repair of DNA fragment ends. PolyA tails and sequencing adapters were added, and PCR amplification was carried out according to the manufacturer’s instructions of the Truseq Nano DNA HT Sample Preparation Kit (Illumina, San Diego, CA, USA). The PCR-amplified products were then purified using the AMPure XP system, initially quantified using Qubit3.0, and the libraries were diluted to 1 ng/μL. The insert size and effective concentration of the libraries were measured using the Agilent2100 Bioanalyzer (Santa Clara, CA, USA) and Applied Biosystems VeritiPro PCR system (Applied Biosystems, Foster City, CA, USA), respectively. The selected libraries were sequenced on the Illumina Hiseq Xten platform (PE150). After re-sequencing, low-quality reads were removed using Trimmomatic (v0.36) [43] to obtain high-quality clean data. The clean reads were aligned to the sheep reference genome (Oar_v1.0) using the BWA (Version 0.7.17) [44] with the command *bwa mem-M*. Subsequently, duplicate reads were marked and removed using SAMBAMBA (Version 0.8.2) [45], and indexing was performed in SAMtools (Version 1.21) [46]. Variant detection was carried out using the GATK (Version 4.3) [42]. The SNPs were filtered using the GATK VariantFiltration protocol with the following settings: FS > 60.0; QD < 10.0; MQ < 40.0; ReadPosRankSum < −8.0; MQRankSum < −12.5. Subsequently, quality control was performed on the resulting SNP dataset (71,403,155 unfiltered SNP sites) using VCFTOOLS (Version 0.1.16) [47] with the criteria of MAF ≥ 0.05, biallelic sites only, genotype missingness < 0.3, and minimum sequencing depth > 3 [22]. After these steps, a total of 23,409,311 SNPs were distributed across 27 chromosomes, and 1150 sheep were obtained for subsequent analysis (N_autosomal SNPs_ = 23,112,008). Meanwhile, we used the PLINK (Version 1.9) [48] indep-pairwise option (indep-pairwise 50, 10, 0.1) to perform linkage disequilibrium (LD) pruning on the SNP dataset to calculate the number of independent SNPs [49] (N = 1,608,328).

### 2.9. Heritability, Genetic Correlation, and GWAS of Rumen Enterotypes

To elucidate the genetic basis of rumen microbial enterotypes, we estimated the heritability of enterotypes and conducted a genome-wide association study (GWAS) based on the genotyping data of the current 1150 Hu sheep. Specifically, first, based on the high-quality genetic variants obtained, we constructed a genetic relationship matrix (GRM) for the 1150 animals. The GRM is calculated using the GCTA (v1.94.1) [50] software with the following equation:

(6) $$g_{ij}=\sum \frac{\left[(x_{ij}-2p_{i})(x_{ik}-2p_{i})\right]}{\left[2p_{i}(1-2p_{i})\right]}$$

where $x_{ij}$ and $x_{ik}$ are the genotypes of individuals i and j, and $p_{i}$ is the allele frequency for the variant in the population. $g_{ij}$ represents the genomic relatedness coefficient between individual i and individual j. Then, we estimated the heritability using a threshold regression model in GCTA (v1.94.1) [50] software. This model assumes the existence of an underlying continuous trait (liability) and that individuals exhibit one of the two binary trait categories when their liability exceeds a certain threshold. Based on this model, we used the *-prevalence* option in GCTA to estimate the heritability of the binary trait on the liability scale. This approach allows for a more accurate calculation of heritability by transforming the estimate from the observed 0–1 scale to the underlying liability scale, which accounts for the disease prevalence. Here, Birthplace and the first five principal components (PCs) of genotypes were included as covariates. The estimation model is as follows:

(7) y=Xb+Wa+e

where y is the vector of observed values of enterotypes (binary traits); b is the vector of fixed effects; a is the vector of additive genetic effects, which follows **a** distribution of N (0, G$\sigma_{a}^{2}$), where G is the GRM and $\sigma_{a}^{2}$ is the additive genetic variance; e is the vector of residual effects, which follows a distribution of N (0, I$\sigma_{e}^{2}$), where I is the identity matrix and $\sigma_{e}^{2}$ is the residual variance. X and W are the incidence matrices for b and a, respectively. The estimated value of h^2^ is $\sigma_{a}^{2}$/$\sigma_{p}^{2}$, where $\sigma_{p}^{2}$ is the phenotypic variance. The likelihood ratio test (LRT) was used to test whether the heritability of a specific phenotype was significant (P_LRT_ < 0.05). We also estimated the genetic correlations ($r_{G}$) of enterotypes and assigned diver genus using a multi-trait model in GCTA v1.94.1 software. The model is consistent with the aforementioned genetic model [7]; however, in this case, y represents the abundance vector of enterotypes or assigned diver genus after log-transformation using the centered log-ratio method. The $r_{G}$ were calculated using the following formula:

(8) $$r_{G}=\frac{{COV}_{G_{XY}}}{\sqrt{\sigma_{G_{X}}^{2}\sigma_{G_{Y}}^{2}}}$$

where $r_{G}$ is the genetic correlation between microbial traits X and Y; ${COV}_{G_{XY}}$ is the genetic covariance matrix of traits X and Y; $\sigma_{G_{X}}$ and $\sigma_{G_{Y}}$ are the genetic standard deviation of traits X and Y.

Regarding the GWAS of enterotypes, we used the generalized linear mixed model (GLMM) in GCTA, which is a GWAS method specifically designed for binary traits and can effectively address the issue of test statistic inflation caused by case–control imbalance. The Birthplace and Season of individuals, as well as the first five eigenvectors of PCA, were used as covariates. Finally, based on the Bonferroni correction, we established the genome-wide significance threshold at *p* < 3.1 × 10^−8^ (0.05/N_independent SNPs, N = 1,608,328) and the suggestive significance threshold at *p* < 6.2 × 10^−7^ (1/N) [51,52,53].The annotation of the variant was taken from the Ensembl Variant Effect Predictor [54]. The frequency distribution of genetic markers in global sheep breeds was investigated using the SheepVar database [55].

### 2.10. The Colocalization Relationship Between Enterotype GWAS Signals and Driving Bacteria GWAS Signals

To evaluate the colocalization relationship between enterotype GWAS signals and driving bacteria GWAS signals, we employed three methods: Overlap Analysis, LD Analysis, and Bayesian Colocalization Analysis. The GWAS for assigned diver genus was previously conducted in our prior study [22], and the summary data from this microbial GWAS were downloaded and utilized for colocalization analysis in the current study. Overlap Analysis: We extracted the suggestively significant SNPs from both the enterotype and driving bacteria GWAS datasets and calculated the number of overlapping significant SNPs. Hypergeometric distribution tests were then employed to evaluate the significance of this overlap. A significantly higher overlap than expected by chance would indicate a substantial overlap between the two GWAS signals. LD Analysis: We extracted the suggestively significant SNPs from both the enterotype and driving bacteria GWAS datasets and used the PLINK tool to compute the LD coefficients (r^2^) between these SNPs. By evaluating the decay of LD, we were able to determine whether the two GWAS signals were closely linked within the genome. Bayesian Colocalization Analysis: We extracted the suggestively significant SNPs from both the enterotype and driving bacteria GWAS datasets and performed Bayesian colocalization analysis using the *coloc* R package (5.2.3) [56]. If the posterior probability for hypothesis H4 (Association with both traits, and a shared SNP) exceeds 0.50, we infer that the two GWAS signals share a common causal variant.

### 2.11. The Influence of Significant Genetic Markers of Enterotype on Rumen Microbiota

To assess the impact of significant genetic markers of enterotypes on the microbial communities in the rumen, we employed a general linear mixed model. In this analysis, we retained only 290 assigned bacterial genera with a prevalence greater than 1.5% [22]. Additionally, for bacterial genera with a prevalence exceeding 60%, we utilized their relative abundance, transformed through centered log-ratio transformation, as quantitative microbial traits; whereas for genera with a prevalence below 60%, their presence/absence binary characteristics were used as binary microbial traits [6,22,57]. Specifically, the model treated microbial traits as the dependent variable, with genetic markers as the primary fixed effect. Moreover, we incorporated birthplace as a covariate to control for potential confounding factors. The model assumed that the dependent variable followed a normal distribution (for relative abundance) or a binomial distribution (for presence/absence states), and the appropriate distribution was selected for fitting based on the specific trait. The final *p*-values were adjusted for FDR using the BH method, with a threshold set at 0.05.

## 3. Results

### 3.1. The Rumen-Enterotypes of the Hu Sheep and Associated Covariates

**Figure 2:**
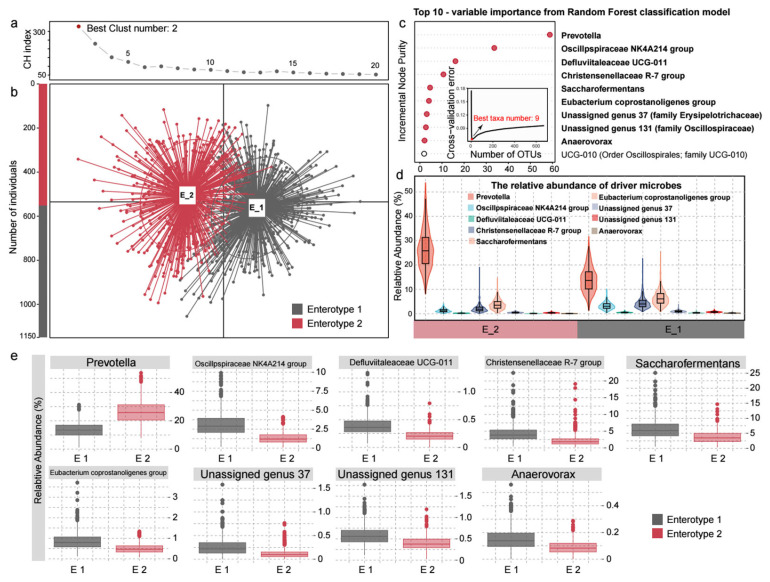
Enterotype Classification of the Rumen Microbiome in 1150 Male Hu Lambs. (**a**) Optimal number of rumen enterotypes separation. The *x*-axis shows the number of enterotypes; the y-axis shows Calinski–Harabasz (CH) index. (**b**) Principal coordinate analysis of the two differential clusters. (**c**) Identification of rumen and rumen enterotypes driver bacteria based on the Random Forest model. (**d**) Overview of the relative abundance of driver bacteria across the two groups. (**e**) A detailed representation of the relative abundance of each driver bacterium across different groups.

For the ruminal microbiota of sheep, the microbial profiling based on the Calinski-Harabasz (CH) index of partitioning around medoids (PAM) revealed that the optimal number of clusters was two (k = 2), indicating that the entire sheep population could be divided into two subgroups based on enterotyping (Figure 2a). These two clusters were designated as Enterotype 1 (E1) and Enterotype 2 (E2). E1 included 597 sheep, while E2 comprised 553 (Figure 2b). A random forest classification model revealed nine driver genera that define these enterotypes, distinguishing a Mixture Enterotype (E1) and a *Prevotella* Enterotype (E2). E2, similar to enterotypes in humans and other mammals [11,23], is characterized by a *Prevotella* dominance (Figure 2c–e). E1, lacking a clear driver genus, is a blend of several genera with higher relative abundances, particularly *Oscillospiraceae NK4A214 group*, *Christensenellaceae R-7 group*, and *Saccharofermentans*.

**Figure 3:**
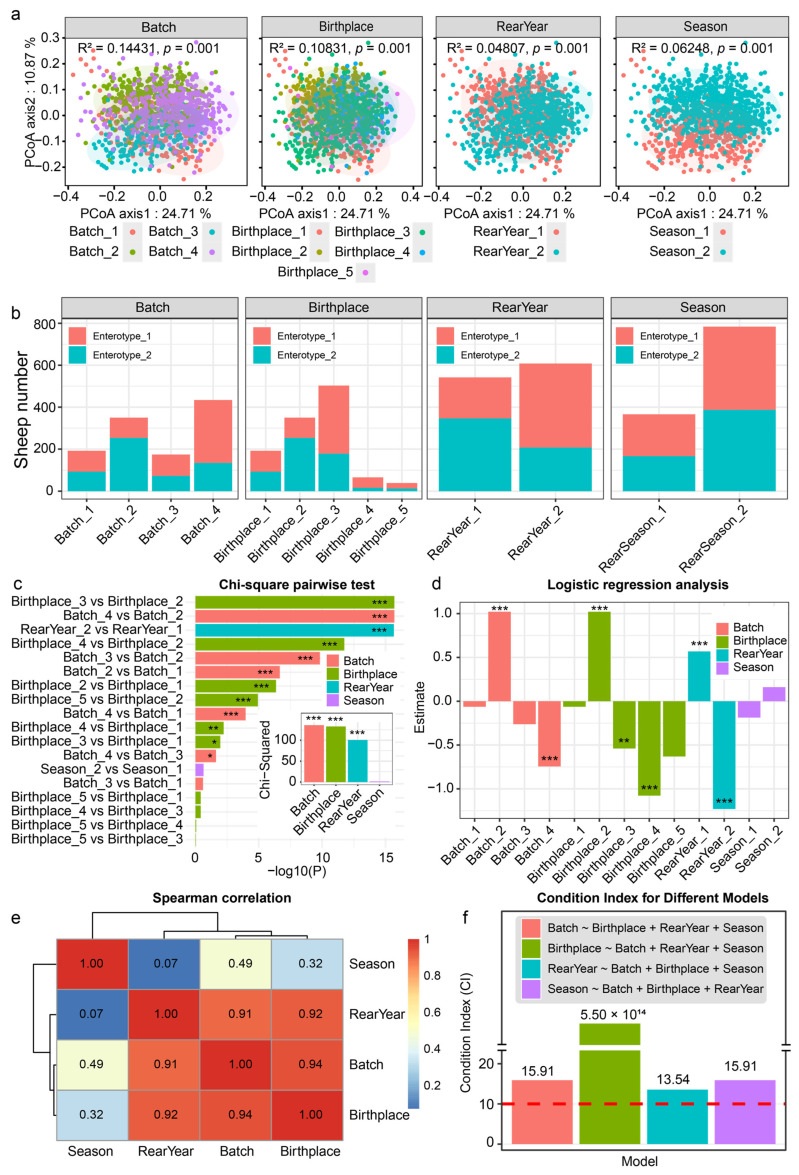
The potential covariates in the sheep population and their impact on the rumen microbial enterotypes. (**a**) The impact of four potential covariates on bacterial community structure at the genus level was assessed using PCOA analysis and PERMANOVA. (**b**) Distribution of different rumen enterotypes of sheep across the four potential covariates. (**c**) Chi-square and pairwise Chi-square tests. (**d**) logistic regression. (**e**) Multicollinearity analysis of covariates based on Spearman’s rank correlation. (**f**) Multicollinearity analysis of covariates based on the condition index (CI). A condition index (CI) value exceeding 10 (represented by the red line) suggests potential multicollinearity between covariates. * *p* < 0.05, ** *p* < 0.01, *** *p* < 0.001.

We further employed PCoA based on Bray–Curtis distance matrices and PERMANOVA to assess the influence of covariates on the overall microbial community structure (Figure 3a). The Batch, Birthplace, Rear year, and Season exerted a significant influence on the microbial community structure at the genus level. Specifically, Batch explained 14.43% of the variance in microbial community structure (R^2^ = 0.1443, F = 64.424, *p* = 0.001); Birthplace accounted for 10.83% (R^2^ = 0.1083, F = 34.771, *p* = 0.001); Rear year explained 4.81% (R^2^ = 0.0481, F = 57.973, *p* = 0.001); and Season explained 6.25% (R^2^ = 0.0625, F = 76.504, *p* = 0.001).

We then conducted chi-square tests and logistic regression analysis to explore the relationship between potential covariates and enterotype (Figure 3b–d, Tables S2–S4). The results revealed significant associations of Batch, Birthplace, and Rear year with enterotype. Specifically, chi-square tests demonstrated that Batch (X^2^ = 136.23), Birthplace (X^2^ = 133.04), and Rear year (X^2^ = 100.69) all significantly influenced enterotype (*p* < 0.001). Pairwise chi-square tests identified significant differences (*p* < 0.05) between various batches, birthplaces, and rearing years (Figure 3c). Logistic regression analysis revealed significant associations between these covariates and enterotype (Figure 3d), with Batch_2, Birthplace_2, and Rear year_1 showing positive correlations. In contrast, Batch_4, Birthplace_3, Birthplace_4, and Rear year_2 were negatively associated with enterotype.

Spearman’s Rank Correlation (Figure 3e) and condition index analyses (Figure 3f) were conducted to examine relationships between covariates and assess multicollinearity, enhancing the stability and interpretability of subsequent regression models. The correlation analysis revealed strong positive associations between Birthplace and Batch (r^2^ = 0.94), Birthplace and Rear year (r^2^ = 0.92), and Batch and Rear year (r^2^ = 0.91), while correlations with Season were weaker (Season-Birthplace r^2^ = 0.32, Season-Batch r^2^ = 0.49, Season-Rear year r^2^ = 0.07). Condition index analysis confirmed that multicollinearity among Birthplace, Batch, and Rear year was significant, with the Birthplace model showing a condition index greater than 30, suggesting severe multicollinearity.

### 3.2. Association of Sheep Performance with Distinct Rumen Enterotypes

**Figure 4:**
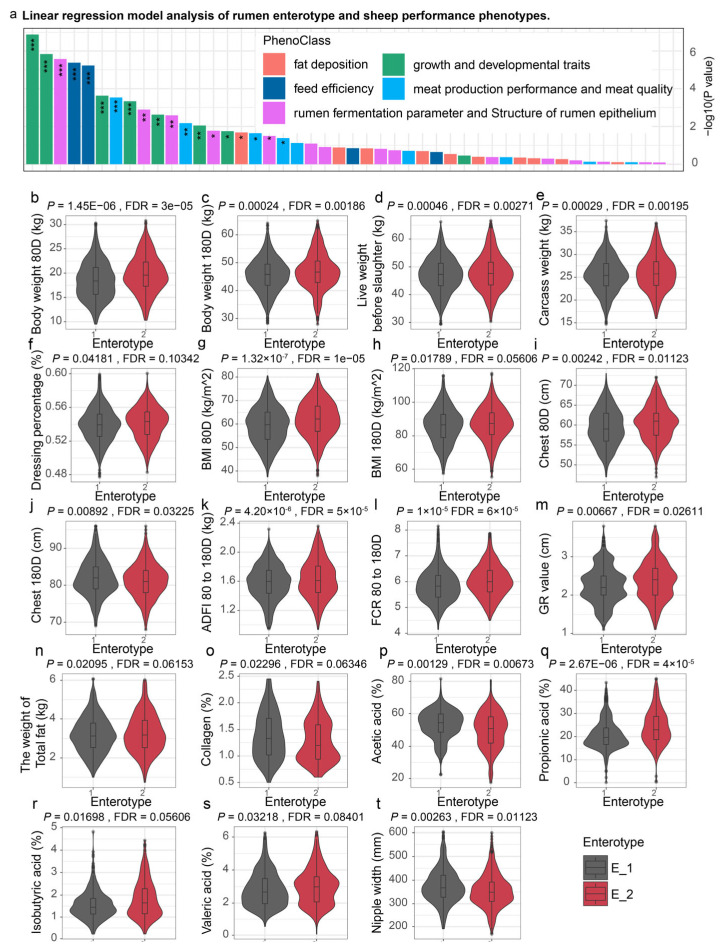
Comparison of productivity traits in sheep of different rumen enterotype. (**a**) The impact of enterotype on sheep performance phenotypes was investigated using a linear regression model, and a stacked plot displays the ranking of *p*-values. (**b**–**t**) The comparison of sheep productivity between different enterotypes. * *p* < 0.05, ** *p* < 0.01, *** *p* < 0.001.

A comprehensive phenotypic comparison was conducted between the E1 and E2 intestinal sheep strains across various traits, including growth development, feed efficiency, fat deposition, rumen fermentation parameters, rumen epithelial structure, and meat quality characteristics (Figure 4 and Table S5).

Firstly, with regard to growth and development, the E2 group demonstrated a significant advantage. At 80 days of age, the E2 group weighed approximately 19.78 kg, significantly higher than the 18.47 kg observed in the E1 group (*p* = 1.45 × 10^−6^). At 180 days of age, the E2 group’s weight was 46.90 kg, compared to 45.64 kg in the E1 group (*p* = 2.38 × 10^−7^). Additionally, the E2 group exhibited superior measurements in chest girth, chest width, and intercostal distance. At 80 days of age, the chest girth in the E2 group was 59.78 cm, whereas the E1 group measured 59.43 cm (*p* = 0.0024). By 180 days, the chest girth of the E2 group was 82.00 cm, surpassing the E1 group’s 81.29 cm (*p* = 0.0089). Furthermore, the E2 group showed enhanced muscle thickness in the chest and back regions, with a muscle thickness of 13.54 mm at 80 days, compared to 13.08 mm in the E1 group (*p* = 0.0757).

Regarding feed efficiency, despite the E2 group demonstrating superior weight and fat deposition, its FCR was 6.03, slightly higher than 5.86 in the E1 group (*p* = 5.95 × 10^−6^). This discrepancy suggests that the E2 group exhibits marginally less efficient energy utilization. The ADG from 80 to 180 days was 0.27 kg for both groups, with no significant difference (*p* = 0.2304), indicating similar growth rates during this phase. Moreover, the E2 group consumed more feed, with an ADFI of 1.63 kg, compared to 1.58 kg in the E1 group (*p* = 4.20 × 10^−6^).

In terms of fat deposition, the E2 group showed significantly higher levels of fat, particularly in tail fat, perirenal fat, and omental fat. The tail fat weight in the E2 group was 1.49 kg, compared to 1.47 kg in the E1 group (*p* = 0.1309). Perirenal fat weighed 0.62 kg in the E2 group, compared to 0.60 kg in the E1 group (*p* = 0.1465). Omental fat in the E2 group was 1.07 kg, slightly lower than the 1.08 kg observed in the E1 group (*p* = 0.2059). These differences in fat deposition reflect a clear advantage in fat storage in the E2 group, with a total fat weight of 3.19 kg, compared to 3.18 kg in the E1 group (*p* = 0.0209).

In terms of meat quality, the E2 group had a lower collagen content (1.28%) compared to the E1 group (1.37%) (*p* = 0.0229), indicating potentially more tender meat in the E2 group. Additionally, the E2 group exhibited slightly higher lean meat and visually detectable fat percentages, at 86.77% and 13.23%, respectively, compared to 85.35% and 14.65% in the E1 group. This difference likely contributes to the superior overall meat quality in the E2 group. In slaughter-related metrics, the pre-slaughter body weight of the E2 group was 47.90 kg, significantly greater than the 47.21 kg observed in the E1 group (*p* = 0.00046), and the post-slaughter carcass weight was 25.95 kg in the E2 group, compared to 25.49 kg in the E1 group (*p* = 0.00029).

Regarding rumen fermentation parameters, the E2 group exhibited significantly higher concentrations of acetic acid and propionic acid. The percentage of acetic acid was 20.44% in the E2 group, versus 18.89% in the E1 group (*p* = 0.0013), while propionic acid percentage was 8.42% in the E2 group, compared to 7.67% in the E1 group (*p* = 2.67 × 10^−6^). However, the butyric acid concentration was 17.26 mmol/L in the E2 group, versus 17.38 mmol/L in the E1 group (*p* = 0.8329), with no significant difference between the groups.

### 3.3. Enterotype-Specific Taxonomic Characteristics in Sheep Rumen Microbiome

**Figure 5:**
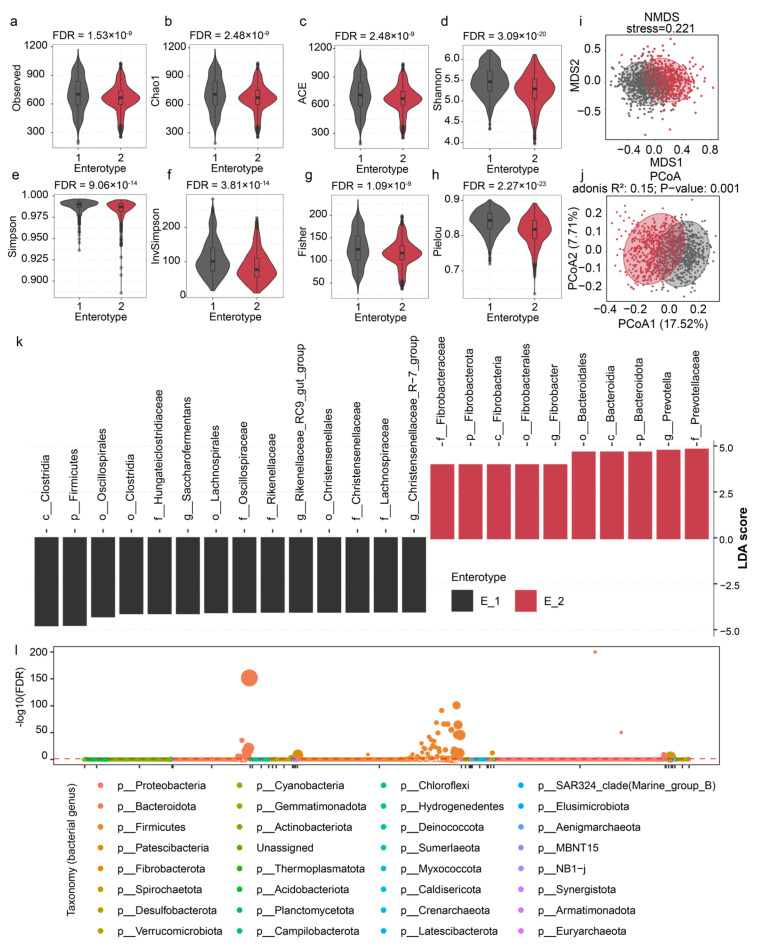
Comparison of differences in α-diversity, β-diversity and rumen microbial composition in different enterotypes. (**a**–**h**) The comparison of rumen microbial α-diversity between different enterotypes. (**i**,**j**) The comparison of rumen microbial β-diversity between different enterotypes. (**k**) Identification of differential rumen microbes between different enterotypes. (**l**) Association analysis of bacterial genera with rumen enterotype.

We compared the diversity and microbial composition between two enterotype populations, revealing higher richness and diversity in E1 compared to E2 (Figure 5a–h and Table S6). The NMDS and PCoA ordinations based on Bray–Curtis distance, as well as PERMANOVA tests, revealed significant differences in the rumen bacterial community structure between the two enterotypes (Figure 5i,j). The LEfSe analysis identified 24 discriminative taxonomic units (LDA > 4, FDR < 0.05) across all taxonomic levels from phylum to species, with 14 enriched in E1 and 10 in E2 (Figure 5k).

At the phylum level, E1 showed higher *Firmicutes* abundance, while *Bacteroidota* and *Fibrobacterota* were less abundant in E2. Family-level analysis revealed higher *Prevotellaceae* and *Fibrobacteraceae* in E2, contrasting with higher *Rikenellaceae* and *Firmicutes* families such as *Christensenellaceae*, *Hungateiclostridiaceae*, *Lachnospiraceae*, and *Oscillospiraceae* in E1. At the genus level, *Prevotella* and *Fibrobacter* were more abundant in E2, whereas *Rikenellaceae RC9 gut group*, *Christensenellaceae R-7 group*, and *Saccharofermentans* were enriched in E1. Notably, *Prevotella*, *Christensenellaceae R-7 group*, and *Saccharofermentans* were the driver genera for the enterotypes.

A two-part association model identified 89 bacterial genera that significantly influenced rumen enterotype (*p* < 0.05; Figure 5l and Table S7), accounting for 10.95% of the total bacterial genera, including 16 unassigned genera. Of these, 75 genera were identified using Beta Regression, while 14 were identified using Zero-Inflated Beta Regression. Even under a more stringent threshold, 68 genera remained, including 14 unclassified genera (FDR < 0.05). Among them, 52 genera had a higher average relative abundance in E1, while 16 genera showed a higher average relative abundance in E2. The cumulative abundance of these 68 genera reached 87.24%, with an average prevalence of 85.20%. Among them, 44 genera were present in the rumen microbiome of at least 1000 sheep. These genera were predominantly from *Firmicutes* (64.71%) and *Bacteroidota* (17.65%).

### 3.4. The Co-Occurrence Network with Its Intrinsic Structure Revealed Enterotype-Specific Differences

**Figure 6:**
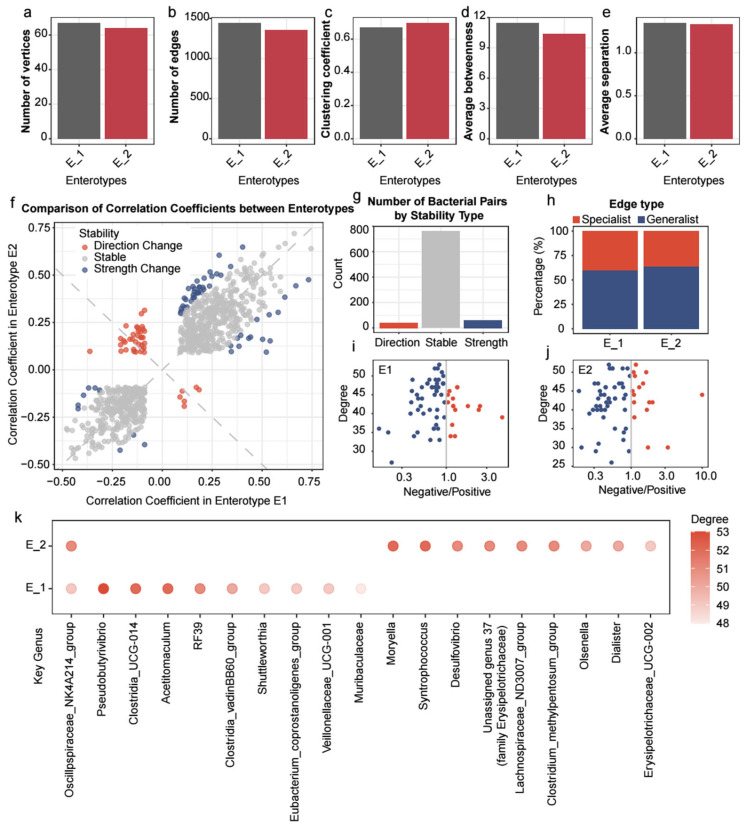
The co-occurrence network with its intrinsic structure revealed enterotypes-specific differences. (**a**–**e**) The topological properties of the cooccurrence networks. (**f**) For the pairs of significant bacterial relationships shared between the two enterotypes (based on Spearman’s rank correlation), we further assessed whether these relationships were stable or altered across the enterotypes. (**g**) The number of pairs for each relationship type. The grey dashed line represents the line of symmetry. (**h**) Proportions of generalist edges and specialist edges in the two enterotypes microbial networks. (**i**,**j**) A scatter plot is presented for each taxon in different enterotypes, showing the log-transformed (log10) ratio of negative to positive interactions against degree. Red nodes indicate that the taxon has more negative interactions than positive interactions, while blue nodes indicate that the taxon has more positive interactions than negative interactions. (**k**) The top 10 key taxa in each of the two enterotypes are represented by colored points, with the color indicating the mean degree of each taxon.

To explore the interactions of rumen microbiota among different enterotypes, we constructed co-occurrence networks at the genus level for both populations. The E1 network had more vertices (n = 67) and edges (n = 1443) compared to E2 (vertices: 64, edges: 1352), and the clustering coefficient of E2 (0.70) was higher than E1, with E2 also showing lower average betweenness and distance (Figure 6a–e).

A comparative analysis of 1931 bacterial correlation pairs revealed that 864 pairs were shared between the two enterotypes, representing common co-occurrence network edges. Among these shared pairs, three correlation patterns emerged: 41 pairs exhibited reversed correlation directions, 61 pairs showed significant strength changes, and 762 pairs had stable correlations across both enterotypes (Figure 6f,g and Table S8). Notably, the correlation between *Christensenellaceae R-7 group* and four other bacterial genera exhibited marked heterogeneity across enterotypes. Similarly, the relationship between *Prevotellaceae UCG-001*, *Saccharofermentans*, and three additional bacterial genera displayed significant fluctuation among the enterotypes (Table 1).

Among the 1931 significant relationships, the remaining 1067 pairs (55.26% of total correlation pairs) were enterotype-specific, being significant in only one of the enterotypes (Figure 6h and Table S6). Specifically, Enterotype 1 exhibited 579 unique relationships (accounting for 40.12% of E1’s total relationships), while Enterotype 2 exhibited 488 unique relationships (representing 36.09% of E2’s total relationships). A chi-square test revealed a statistically significant difference in the number of unique and shared edges between the two enterotypes (Chi-square test *p*-value: 0.03). Figure 6k shows the 10 vertices with the highest degree in each network (a total of 19 genera). *Oscillpspiraceae NK4A214 group* is a shared key microbe in the co-occurrence networks of both enterotypes. We further analyzed the positive and negative correlations of each genus in each enterotype network (Figure 6i,j). Interestingly, in both enterotypes, the majority of genera have more positive correlations (Pos_E1:51; Pos_E2:48) with other genera than negative correlations (Neg_E1:16; Neg_E2:16). Only a few genera have more negative correlations than positive correlations with other microbes, including *Dialister*, *Erysipelotrichaceae UCG-002*, *Olsenella*, and *Syntrophococcus* in the key taxa of E2. Notably, no negative correlations were observed in the key taxa of E1.

### 3.5. GWAS Identifies Several Genomic Variants Affecting Enterotype

Genetic estimates derived from the threshold model indicated that the proportion of phenotypic variance attributable to genetic variance, namely heritability, was 0.27, with a standard error of 0.10. The heritability on the liability scale was estimated to be 0.43, with a standard error of 0.1517. The likelihood ratio test confirmed the significance of these estimates with a *p* value of 4.3869 ×10^−4^. We estimated the genetic correlation between enterotype and taxonomically defined driving bacteria using a multi-trait animal model. The results revealed significant differences in the genetic correlations between enterotype and driving bacteria. For example, the genetic correlation between enterotype and *Prevotella* was −0.86 (SE = 0.09), indicating a significant negative correlation, meaning that changes in enterotype were inversely related to the abundance of *Prevotella*. In contrast, the genetic correlations between enterotype and *Oscillospiraceae NK4A214 group*, *Defluviitaleaceae UCG-011*, *Christensenellaceae R-7 group*, *Saccharofermentans*, *Eubacterium coprostanoligenes group*, and *Anaerovorax* were 0.96 (SE = 0.10), 0.93 (SE = 0.19), 0.94 (SE = 0.07), 0.61 (SE = 0.19), 0.61 (SE = 0.15), and 0.82 (SE = 0.24), respectively, all showing significant positive genetic correlations.

**Figure 7:**
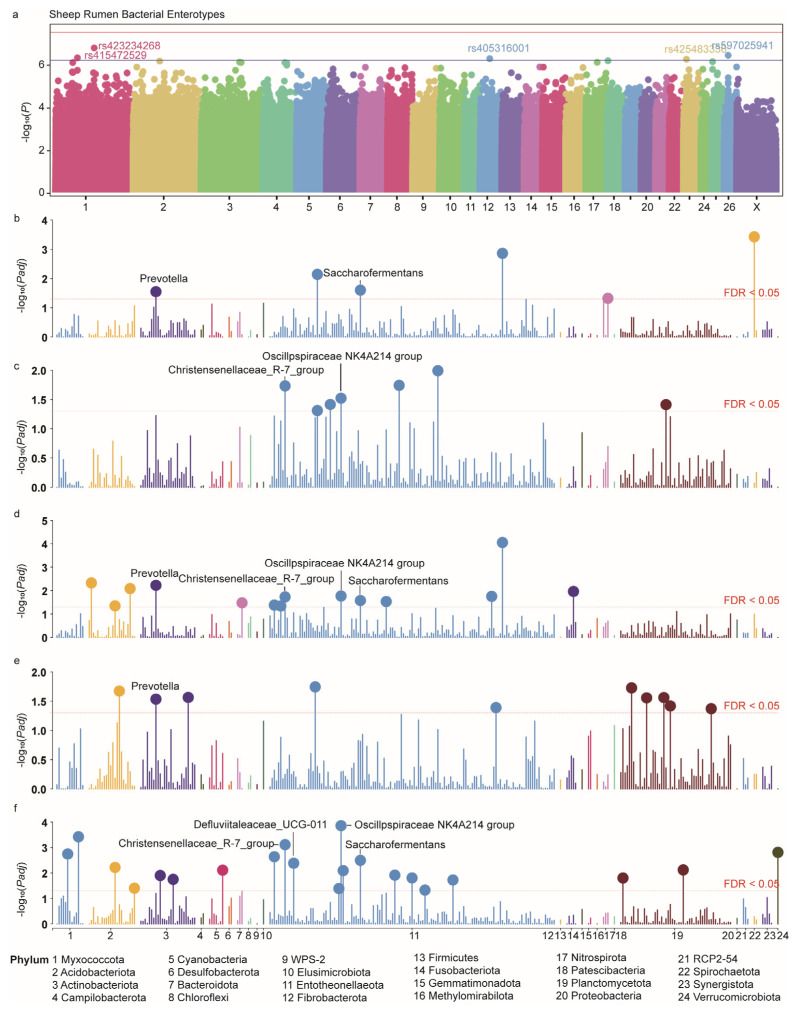
Enterotype-related genetic variations and their effects on rumen microbiota. (**a**) Manhattan plot of enterotypes-GWAS results. (**b**–**f**) The impact of seven SNPs associated with enterotypes on rumen microbial genus investigated using linear modeling.

We further conducted a GWAS analysis using a generalized linear mixed model suitable for binary traits to identify host genomic variants influencing rumen enterotypes. A total of five genome-wide significant genetic markers were identified, located on chromosomes 1 (rs415472529, rs423234268—an intronic variant in the first intron of the *CHODL* gene), 12 (rs405316001), 23 (rs425483330), and 26 (rs597025941—an intronic variant in the first intron of the *ENPP6* gene) at the whole-genome-suggestive significance threshold (Bonferroni-adjusted; Figure 7a and Table S10). The mean minimum allele frequency of these SNPs in this population is 0.16, with a minimum of 0.09 and a maximum of 0.39. No genomic inflation was observed in the GWAS (lambda = 1.004).

### 3.6. Enterotype-Related Genetic Variations and Their Effects on Rumen Microbiota

We applied Overlap Analysis, Linkage Disequilibrium Analysis, and Bayesian Colocalization Analysis to examine whether enterotype GWAS signals and driving bacteria GWAS signals share the same genetic variation. No significant colocalization or overlap was observed, and the LD analysis revealed an extremely low average r^2^ of 0.001 between significant SNPs from both GWAS datasets. Bayesian colocalization further corroborated this finding, as the posterior probability did not exceed the 0.50 threshold.

In the absence of colocalized loci, we further compared the differences in microbial genera associated with the genotypes of the aforementioned SNPs to investigate whether genetic variation influences the rumen microbial enterotype by modulating the driving bacteria, as well as the broader bacterial genera affected by these loci. These genetic markers significantly impacted 58 bacterial taxa, including five driver bacteria (FDR < 0.05; Figure 7e,f and Table S11). *Prevotella*, *Oscillospiraceae NK4A214 group*, *Christensenellaceae R-7 group*, and *Saccharofermentans* exhibited significantly different abundances among different genotypes of three significant SNPs. The *Defluviitaleaceae UCG-011* demonstrated significant abundance variations associated with one distinct significant SNP.

## 4. Discussion

With the development of metagenomic sequencing technologies based on bacterial DNA analysis, there has been a qualitative leap in our understanding of the gut microbiota. The gut microbiota exhibits significant individual variation over time and space, which has posed challenges to advancing our understanding of the complex biological relationships between the host and its gut microbiome. The introduction of the enterotype concept marked the first step toward dimensionality reduction analysis of the gut microbiota in both humans and animals, providing a groundbreaking framework for studying host–microbe interactions. Over a decade since its conceptualization, rumen enterotype research in ruminants has attracted growing interest [18,58], yet remains critically underexplored in sheep. To our knowledge, this represents the first large-scale multi-omics study to characterize rumen enterotypes in sheep and establish their genetic basis and multi-trait productivity associations. Here, we identified two enterotypes with distinct microbial signatures and interaction networks in 1150 sheep, revealing their profound impacts on host development, feed efficiency, meat quality, rumen fermentation, and other productive traits.

We identified a *Prevotella*-dominated enterotype (E2) that demonstrates cross-species conservation with enterotypes previously reported in humans [59], Holstein cattle [58], and goats [60]. While this enterotype shows genus-level conservation across species, it is important to note that distinct strains of *Prevotella* may be present in different host species. The enrichment of cellulolytic functions in E2 underscores *Prevotella*’s central role in plant polysaccharide metabolism [61]. In contrast to the *Ruminococcus*-driven enterotypes in prior studies, E1 represents a novel “mixed enterotype” co-dominated by *Christensenellaceae R-7 group*, *Saccharofermentans*, and *Lachnospiraceae NK3A20 group*. This finding parallels observations from a large-scale human gut study (n = 2678), where mixed enterotypes predominated [62], suggesting that larger cohorts enhance detection of functionally redundant microbial consortia [63]. Such redundancy, driven by convergent metabolic pathways, may obscure signals from individual biomarker taxa [64]. Furthermore, while defining enterotypes and aggregating bacteria into genera can improve the interpretability of microbial community data, it may also lead to a loss of finer-scale functional diversity. In fact, different strains within the same bacterial genus can exhibit highly diverse functions, which may be masked by genus-level aggregation. For instance, despite lacking a dominant taxon, E1’s enrichment of *Ruminococcus* and *Acetitomaculum* showed striking overlap with bovine mixed enterotypes [18]. Moreover, *Lachnospiraceae NK3A20 group* abundance positively correlated with rumen papilla development (r = 0.32, *p* < 0.01), likely via butyrate-mediated epithelial morphogenesis [65,66]. This “functional modularity” confers ecological resilience to mixed enterotypes in maintaining host homeostasis yet complicates targeted microbial manipulation.

Enterotype divergence directly shaped host phenotypic outcomes: E2 sheep achieved superior growth performance and meat yield, potentially mediated by *Prevotella*’s enhanced fiber degradation [67,68]. However, these outcomes were associated with reduced feed efficiency and excessive adiposity, which could be a consequence of energy being redirected toward fat storage. Since the accretion of fat tissue requires more energy than muscle tissue, higher levels of adiposity may therefore impair feed efficiency. Notably, E2 sheep also consumed more feed compared to E1 sheep, which may contribute to the observed differences in both growth and adiposity. Differences in feed intake can influence rumen retention time and microbiota composition, further complicating the direct attribution of phenotypic outcomes to microbiota alone. Longitudinal studies that track feed intake, rumen retention time, and microbiota dynamics across different enterotypes would help clarify the causal relationships between these factors [69]. Conversely, E1’s enrichment of *Christensenellaceae* (associated with leanness phenotypes in humans [70]) and *Saccharofermentans* [71] likely enhanced feed utilization via optimized energy partitioning, yet constrained rapid weight gain potential [72]. Network analysis revealed fundamental structural differences: The E1 network exhibited greater scale (67 nodes, 1443 edges) with predominantly synergistic interactions (e.g., acetate synthesis coupling between *Acetitomaculum* and *Pseudobutyrivibrio*) [73], while E2 showed higher integration (clustering coefficient = 0.70) but contained competitive interactions (e.g., *Dialister*), possibly reflecting niche competition for starch substrates [74]. These structural disparities suggest that E1 microbiota buffer environmental fluctuations through functional redundancy, whereas E2 communities prioritize metabolic efficiency via specialized niche partitioning [64,75].

Our results demonstrate that the host genome exerts a statistically significant influence on the composition of the rumen microbiota, a finding that is consistent with emerging evidence suggesting that host genetics contribute to the shaping of the rumen microbial ecosystem in ruminants [22,76,77]. The identification of five genome-wide suggestive significant SNPs and two candidate genes provides mechanistic insights into this relationship. Notably, ENPP6 encodes a phospholipase involved in lipid metabolism, potentially modulating rumen pH or nutrient availability to favor specific bacterial taxa like *Saccharofermentans* and *Christensenellaceae R-7 group* [78,79,80]. Similarly, *CHODL* are implicated in cellular differentiation and immune regulation, suggesting their roles in creating host-specific niches for driver bacteria such as *Prevotella* and *Oscillospiraceae NK4A214 group* [77,81,82]. Our observation that genetic variants alter abundances of 58 bacterial taxa, including key functional guilds, supports the hypothesis that host genetics indirectly shapes microbial communities by modifying the physicochemical or immunological rumen environment [83]. These results advance our understanding of host–microbiome coevolution and highlight genetic markers for future breeding strategies targeting rumen efficiency. This host–microbe co-evolutionary mechanism unveils novel targets for marker-assisted selection: breeding individuals with “ideal enterotype” genotypes could synergistically enhance host genetic potential and microbiome functionality. However, caution is warranted: environmental confounders (e.g., diet, geography) may perturb enterotype classification, and whether functional redundancy coincides with metabolomic divergence remains unvalidated. Future studies should employ rumen microbiota transplantation to establish causal enterotype–phenotype relationships, coupled with metabolomics to dissect the molecular basis of functional equivalence.

## 5. Conclusions

Our comprehensive analysis of the rumen microbiome across 1150 sheep revealed two distinct enterotypes with different microbial compositions and functional characteristics. This is the first study to identify enterotypes in sheep. Enterotype 1 was defined by a mixture of several genera, while Enterotype 2 was dominated by *Prevotella*. These enterotypes were associated with significant differences in sheep performance, particularly in growth, feed efficiency, meat production, and ruminal fermentation parameters. E2 sheep exhibited superior growth and meat production but lower feed efficiency and increased fat deposition. The heritability of enterotypes, estimated using a threshold model, was found to be 0.43. A GWAS subsequently identified five significant genetic markers associated with rumen enterotypes, influencing 58 bacterial taxa, including several key and driving taxa. The personalized rumen microbiome enterotypes and functional gene markers identified in this study provide novel intervention targets for enhancing sheep production performance through targeted modulation of the gut microbiota. These findings not only deepen our understanding of the rumen microbiome ecosystem in sheep but, more importantly, uncover highly actionable microbial regulation sites, thereby laying the theoretical foundation for the development of precise microbiome-based intervention strategies.

## Figures and Tables

**Table 1:** Bacterial genus correlation pairs with both direction and strength changes across different enterotypes.

Taxa_A	Taxa_B	r (E1)	r (E2)
*Acetitomaculum*	*Shuttleworthia*	−0.21	0.13
*Butyrivibrio*	*Eubacterium nodatum group*	−0.13	0.09
*Christensenellaceae R-7 group*	*Anaerovibrio*	−0.21	0.16
*Christensenellaceae R-7 group*	*Eubacterium ruminantium group*	−0.10	0.21
*Christensenellaceae R-7 group*	*Pseudobutyrivibrio*	−0.10	0.23
*Christensenellaceae R-7 group*	*Veillonellaceae UCG-001*	−0.18	0.23
*Clostridia UCG-014*	*Acetitomaculum*	−0.10	0.30
*Defluviitaleaceae UCG-011*	*Veillonellaceae UCG-001*	−0.12	0.18
*Eubacterium coprostanoligenes group*	*Anaerovibrio*	−0.21	0.10
*Eubacterium ruminantium group*	*NK4A214 group*	−0.09	0.12
*F082*	*Fibrobacter*	−0.15	0.16
*F082*	*Saccharofermentans*	−0.13	0.23
*Fibrobacter*	*Lachnospiraceae ND3007 group*	−0.09	0.14
*Oribacterium*	*Veillonellaceae UCG-001*	0.11	−0.19
*Prevotellaceae Ga6A1 group*	*Lachnospiraceae XPB1014 group*	−0.14	0.11
*Prevotellaceae UCG-001*	*Desulfovibrio*	0.10	−0.11
*Prevotellaceae UCG-001*	*NK4A214 group*	−0.19	0.15
*Prevotellaceae UCG-001*	*Succiniclasticum*	0.11	−0.17
*Prevotellaceae YAB2003 group*	*Eubacterium ruminantium group*	0.09	−0.14
*Pseudobutyrivibrio*	*UCG-010*	−0.12	0.20
*RF39*	*Acetitomaculum*	−0.16	0.16
*RF39*	*Succiniclasticum*	−0.14	0.10
*Rikenellaceae RC9 gut group*	*Eubacterium ruminantium group*	−0.18	0.11
*Rikenellaceae RC9 gut group*	*Saccharofermentans*	−0.11	0.20
*Ruminococcus gauvreauii group*	*probable genus 10*	−0.09	0.31
*Saccharofermentans*	*Dialister*	0.17	−0.09
*Saccharofermentans*	*Pseudobutyrivibrio*	−0.13	0.15
*Saccharofermentans*	*Veillonellaceae UCG-001*	−0.36	0.10
*UCG-004*	*Anaerovibrio*	−0.15	0.19
*UCG-004*	*Pseudobutyrivibrio*	−0.12	0.13
*UCG-010*	*Veillonellaceae UCG-001*	−0.09	0.20
*Veillonellaceae UCG-001*	*Candidatus Saccharimonas*	−0.10	0.17

r: Correlation coefficient.

## Data Availability

Individual-level raw data including host genetics and 16S rRNA sequencing data have been uploaded to the Genome Sequence Archive (GSA) database (https://ngdc.cncb.ac.cn/gsa/ (accessed on 30 August 2025)). Whole genome resequencing data numbers are CRA019576 and CRA019589, and 16S rRNA sequencing data accession number is CRA019574.

## Supplementary Materials

The following supporting information can be downloaded at https://www.mdpi.com/article/10.3390/ani15182724/s1. Table S1. Diet information for animal experiments (air-dry basis); Table S2. The number of sheep with different rumen bacterial enterotypes within different covariates; Table S3. Pairwise chi-square tests are used to compare the significance of proportions between different categories within covariates; Table S4. Logistic regression analysis is used to compare the impact of different categories within covariates on rumen bacterial enterotypes; Table S5. Linear Regression Analysis of Enterotype Effects on Sheep Performance Phenotypes; Table S6. Linear Regression Analysis of Enterotype Effects on Rumen Microbiome Alpha Diversity in Sheep; Table S7. Impact of Enterotypes on 813 Bacterial Genera in Sheep Rumen: A Two-Part Beta Regression Approach; Table S8. Comparative Analysis of Significant Spearman’s Rank Correlation Pairs in Two Enterotypes: Shared Pairs and Their Changes; Table S9. Distribution of Significant Spearman’s Rank Correlation Pairs Across Two Enterotypes; Table S10. Genome-Wide Association Study (GWAS) of Enterotypes in Sheep Rumen Microbiota: Significant SNP Associations; Table S11. FDR Values for Linear Regression Models Assessing the Effects of Enterotype-GWAS Significant SNPs on Rumen Bacterial Genera.

## Abbreviations

The following abbreviations are used in this manuscript:

E1	Enterotype 1
E2	Enterotype 2
INRAE	Institut National de Recherche pour l’Agriculture, l’Alimentation et l’Environnement
RF	Random Forest
GWAS	Genome-wide association studies
VFA	Volatile fatty acids
PCA	Principal Component Analysis
DMI	Dry matter intake
BMI	Body mass index
FI	Feed intake
ADFI	Average daily feed intake
FCR	Feed conversion ratio
ADG	Average daily gain
MBW	Mid-test metabolic weight
RFI	Residual feed intake
GR	Greville
EMA	Area of the eye muscle
ACE	Abundance-based Coverage Estimator
CI	Condition index
PCoA	Principal coordinate analysis
NMDS	Non-metric multidimensional scaling
FDR	False discovery rate
BH	Benjamini–Hochberg
ZIBR	Zero-Inflated Beta Regression
SNP	Single nucleotide polymorphism
MAF	Minor Allele Frequency
LD	Linkage disequilibrium
REML	Restricted maximum likelihood
PCs	Principal components
GRM	Genomic relationship matrix
LRT	Likelihood ratio test
GLMM	Generalized linear mixed model
LEfse	Linear Discriminant Analysis Effect Size
LDA	Linear Discriminant Analysis
PERMANOVA	Permutational multivariate analysis of variance
CH	Calinski–Harabasz
PAM	Partitioning around medoids
